# Identification of Novel Progesterone Receptor (PR) Inhibitors (*Homo sapiens*) from Metabolites of Biotransformation Fungal: A Bioinformatics Approach

**DOI:** 10.3390/ph18020136

**Published:** 2025-01-21

**Authors:** Janaína de Araújo E. Dourado, Samuel Q. Lopes, David Esteban Q. Jimenez, Ryan S. Ramos, Irlon M. Ferreira

**Affiliations:** 1 Laboratório de Biocatálise e Síntese Orgânica Aplicada, Departamento de Ciências Exatas e Tecnológicas, Universidade Federal do Amapá, Macapá 68902-280, Brazil; janainae757@gmail.com (J.d.A.E.D.); samuql12@gmail.com (S.Q.L.); derteriom@gmail.com (D.E.Q.J.); 2 Programa de Pós-Graduação em Ciências Farmacêuticas, Departamento de Ciências Biológicas e da Saúde, Universidade Federal do Amapá, Macapá 68902-280, Brazil; 3 Laboratory of Modeling and Computational Chemistry, Department of Biological and Health Sciences, Federal University of Amapá, Macapá 68902-280, Brazil; ryanquimico@hotmail.com

**Keywords:** steroids, fungi, ADMET, androstenedione, hydroxyprogesterone, dihydrotestosterone

## Abstract

**Background/Objectives**: Steroids have demonstrated selective cytotoxic properties against tumor cells. The pro-gesterone receptor (PR) plays a vital role in the proliferation, cell differentiation, and maintenance of female reproductive tissue, and its malfunction can lead to breast cancer. The use of the biocatalytic method by filamentous fungi has sparked interest in the obtained of steroids due to the advantages of the process. **Methods:** Pharmacokinetic and toxicological properties (rat and mouse), molecular docking simulation studies, and prediction of the spectrum of biological activity were performed to select molecules with the potential for PR inhibition, from 155 biotransformed products of the progesterone. Subsequently, the chemical structures were subjected to an evaluation of their pharmacokinetic and toxicological properties and, with the application of ADMET filters. **Results:** Androstenedione, 17α-hydroxyprogesterone, and dihydrotestosterone, obtained by the process of biotransformation of PR by different filamentous fungi, showed good pharmacokinetic profiles and low toxicity compared to the control groups. The in-silico data associated with molecular docking studies revealed the best binding affinity and similarity in the interactions of these molecules against the human progesterone receptor target. Thus, the results of biological activity spectrum prediction highlight the great potential to investigate the role of molecular descriptors in the attribution of anti-cancer activities. **Conclusions:** The biocatalytic process, by filamentous fungi, can provide important molecules as a product of progesterone biotransformation, such as androstenedione, 17α-hydroxyprogesterone, and dihydrotestosterone. In this study we showed that these molecules have good pharmacokinetic profiles and low toxicity for antineoplastic activity (breast cancer).

## 1. Introduction

Breast cancer (BC) is a leading cause of death worldwide, and its high incidence is often linked to the expression of hormone receptors such as the progesterone receptor (PR) [1,2]. This receptor plays a critical role in cell proliferation and differentiation in breast tissue, and its dysregulated expression [3] may contribute to the development and progression of malignant tumors. Several PR-targeted therapies are currently in use. However, conventional drugs, such as selective progesterone receptor modulators, have significant side effects, including tumor resistance and systemic toxicity, which limit their long-term efficacy and safety [4].

In 2024, 2,001,140 new cancer cases and 611,720 cancer deaths are projected to occur in the United States. Cancer mortality continued to decline through 2021, averting over 4 million deaths since 1991, because of reductions in smoking, earlier detection for some cancers, and improved treatment options in both the adjuvant and metastatic settings. However, these gains are threatened by increasing incidence for 6 of the top 10 cancers [5]. This index indicates that BC is the most prevalent form of cancer worldwide, surpassing lung cancer in several cases [6]. The high incidence and significant impact on public health highlights the importance of developing new therapeutic approaches, especially those with lower toxicity and greater long-term efficacy.

Therefore, the search for new compounds with potential anticancer activity and fewer side effects is a priority. Compounds derived from steroids, which are normally difficult to produce by traditional synthetic methods, can be effectively produced by microbial transformation [7]. The studies being conducted in this area were started in 1950 when the pharmacological properties of progesterone and cortisol were announced, and when a *Rhizopus* species’ 11α-hydroxylation activity was identified. This was a pivotal moment in the creation of steroids with beneficial biological actions for commercial production [8], and it is widely acknowledged that microbial systems can produce pharmaceutical industry-useful steroids [9,10]. The use of microorganisms from the environment, especially filamentous fungi, has sparked interest in the biotransformation of steroids due to the great diversity of species, consequently generating a vast library of steroid products obtained by this method [11].

These steroids have demonstrated selective cytotoxic properties against tumor cells and have been explored for their potential as anticancer agents [12]. Among the recent advances, bioinformatics studies have facilitated the screening and optimization of new drug candidates by providing a detailed analysis of the molecular interactions and binding affinities between fungal-derived compounds. These computational approaches aid in the rapid identification of potential PR-specific inhibitors, thereby reducing the cost and time associated with initial experimental trials [13].

The search for new drugs also considers the need to overcome the adverse effects of the drugs currently in use, such as tumor resistance and endocrine side effects. Thus, the combination of experimental exploration of the biotransformation of progesterone by fungi and in silico studies may offer promising and potentially less toxic therapeutic alternatives for the treatment of hormone-dependent BC. The current study aimed to identify novel PR inhibitors (*Homo sapiens*) from the products of progesterone biotransformation by different fungal species using bioinformatics.

## 2. Results and Discussion

### 2.1. Predictions of Pharmacokinetic and Toxicological Properties

**Figure 1:**
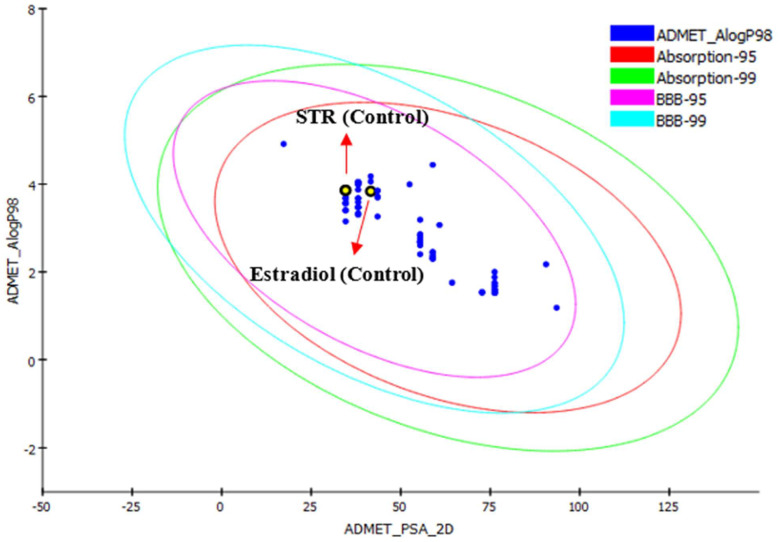
Pharmacokinetic predictions of the top 91 molecules (Alogp98 vs. PSA_2D).

Molecules identified by biotransformation by fungal species were subjected to a pharmacokinetic study involving 115 chemicals. The molecules were subjected to five pharmacokinetic filters, resulting in 91 molecules (Figure 1) within the pharmacokinetic acceptance range (ellipse models). STR and estradiol were used as controls because they are marketed for the treatment of ovulation disorders associated with hormone deficiency, such as pain and other menstrual cycle changes, secondary amenorrhea, benign breast changes, and vulvovaginal atrophy.

Parameters related to absorption, distribution, metabolism, and excretion (ADME) are highly correlated in the development of new drugs, as a prior study of pharmacokinetic properties increases the probability of the drug candidate passing the later phases [14]. Pharmacokinetic descriptors for the TopHits3 molecules were thus obtained, which represented good descriptors of oral bioavailability, penetration of the blood–brain barrier (BBB), hepatotoxicity, human intestinal absorption (HIA), plasma protein binding (PPB), solubility (water solubility), and binding to cytochrome P450 2D6 (CYP2D6) (Table 1).

Before performing ligand–receptor interaction studies (molecular docking simulations), it is essential to know the properties of a molecule, such as (i) its physicochemical properties, (ii) drug similarity or bioactivity/oral bioavailability scores, (iii) ADMET, and (iv) toxicity [14]. All pharmacokinetic and toxicological parameters must follow the standardized Lipinski rule (rule of five or R5), which is available for the determination of such properties.

Regarding the ADMET analysis of the 115 molecules, 3 (TopHists3) stood out in comparison to the control groups owing to their good pharmacokinetic profile, that is, no hepatotoxicity, ease of binding to plasma protein, good aqueous solubility, intestinal absorption, and high penetration in the BBB (Table 1).

**Figure 2:**
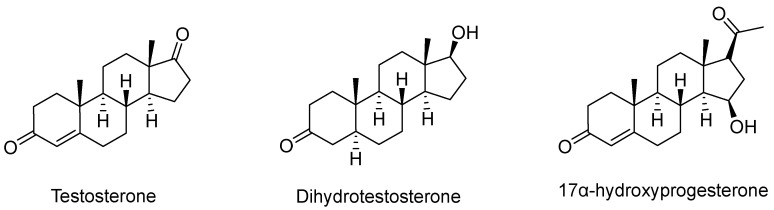
Molecular structure (2D) of the TopHits3 compounds with low risk of toxicity.

In a standard toxicity risk evaluation, the TOPKAT 6.2 software locates fragments within the molecular structure with a toxicity risk based on U.S. Food and Drug Administration (FDA) descriptors. The results of the toxicological predictions for the TopHits3 molecules are shown in Figure 2, Table 2, and Tables S1 and S2 in the Supplementary Materials.

The Ames test is a reversible bacterial mutation assay that simulates mammalian metabolism and evaluates genetic damage and frameshift mutations in the presence of a chemical [16]. The TopHits3 molecule prediction showed that none of the chemicals were mutagenic, i.e., they did not induce genetic errors. However, a multi/single alert for carcinogenicity was observed for all molecules in the mouse model. Concerning the dose values for RMTD, the chemicals androstenedione and dihydrotestosterone presented values between 0.046 and 0.064 g/kg, lower than those of the STR and estradiol controls.

15α-Hydroxyprogesterone, a commercial compound, had a higher RMTD (0.094 g/kg) than the STR control. Thus, it is a candidate drug because it presents fewer adverse effects for carcinogenic potency and a low risk of toxicity in animal models (rats) with higher LD50 and LOAEL dose values. In aquatic organisms (*Pimephales promelas* and *Daphnia magna*), it presents higher LC50 and EC50 values than the STR control.

The carcinogenic potential of progestogens and associated therapies have been extensively reviewed by the IARC [17]. The individual compounds medroxyprogesterone acetate (MPA), megestrol acetate, and chlormadinone acetate induce tumors in the mammary glands of dogs, whereas norethisterone and norethynodrel induce hepatocellular adenomas or carcinomas in rats. In female mice, MPA induces mammary adenocarcinomas after subcutaneous administration [18], whereas norethisterone induces only benign ovarian tumors. In addition, norethisterone increased the incidence of malignant mammary tumors in rats, whereas the incidence of combined adenomas and carcinomas was increased by norethisterone only in male rats.

The carcinogenic effects of progestogens are complex and have not been fully elucidated. The available evidence suggests that progestogens and their derivatives are non-genotoxic carcinogens. This effect is believed to occur primarily via a receptor-mediated mechanism. PR ligation is associated with cell proliferation in normal human mammary epithelial cells through the indirect production of paracrine growth factors [19], as well as in rodent and/or human mammary tumor cells with MPA [20,21].

Evidence regarding the binding of 17-β-estradiol to the estrogen receptor has been extensively reviewed [22]. The receptor appears to bind the ligand, and as a result, all four rings of the steroid core contribute significantly to binding. In addition, hydrogen bonding between the receptor and the phenolic hydroxyl groups and 17-β also plays a role. Binding is generally reduced by introducing polar substituents into the structure, whereas hydrophobic groups are tolerated at various positions and are subject to steric constraints. In vitro receptor binding assays suggest that the phenolic hydroxyl group is more important than the 17-β hydroxyl group in terms of the estrogen receptor binding affinity [23].

This alert describes the teratogenicity of androgenic steroids and androgen receptor (AR) agonists with significant fetal virilization potency in vivo. In addition to primary androgens such as androstenedione, other compounds such as androgen-derived progestins (synthetic progesterone) with residual or secondary androgenic properties [24] are also included.

The presence of a structural alert for skin sensitization or irritation caused by a molecule indicates its potential to cause skin sensitization. Whether a molecule is a skin sensitizer depends on its percutaneous absorption. Generally, small lipophilic molecules are more easily absorbed by the skin and are therefore more likely to cause sensitization. Consequently, all molecules exhibited a moderate alert for skin irritation, with only molecule 70 displaying a severe alert for eye irritation.

### 2.2. Molecular Docking

Computational methods have emerged as new approaches for evaluating the therapeutic potential and elucidating the biological mechanisms of medicinal plants and molecules originating from fungal and bacterial biotransformation processes [15]. Molecular docking is used to select protein targets of pharmacological interest and identify potentially specific interactions of these targets. Computational studies have a high potential for drug repositioning simulations, target identification, ligand–receptor interaction or inhibition profiling, and the construction of biological models with high similarity to experimental models.

The validation results of the molecular docking simulation protocols were considered satisfactory because the relative overlay (pose plus orientation plus torsion) of the crystallographic ligand (control—experimental model) and the docked ligand (docking pose—theoretical model) were similar. In the human PR recovering its pose, it was possible to validate the molecular docking protocols after calculating the root mean square deviation (RMSD), that is, the measurement of the average distance between the atoms of the two molecules, which was 0.58Å and ΔG = −11.92 kcal/mol. Thus, the literature specifies that when RMSD values ≤ 2Å, the docking protocol is considered satisfactory because it is like the experimental model [25,26,27].

**Figure 3:**
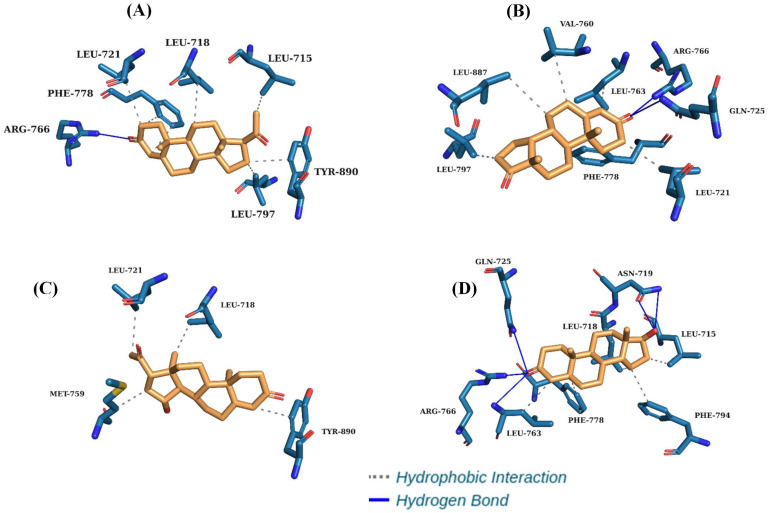
Three-dimensional interactions of the human progesterone receptor binding site with STR molecules (**A**), androstenedione (**B**), 15α-hydroxyprogesterone (**C**), and dihydrotestosterone (**D**).

The pose obtained from the molecular docking simulation allowed us to conclude that STR binds to amino acid residues in the binding site of the human PR, like the crystallographic pose. In the active site, the observed interactions are in the α-helix between the amino acid residues Leu-715, Leu718-Leu721, Leu797, and Tyr890 and the β-sheet between amino acid residues Met775–Phe778. In the STR, hydrogen bonding was observed in the α-helix with the residue Arg766. The interactions obtained by molecular docking (Figure 3) agreed with the results of Williams and Sigler [28], who evaluated the atomic structure of progesterone complexed with its receptor. Arg766 plays a critical supporting role, as highlighted by its conservation in the corresponding sequence position in all steroid receptors.

The PR, a member of the nuclear receptor (NR) superfamily, plays a vital role in the development, differentiation, and maintenance of female reproductive tissues. The binding of PR ligands such as progesterone induces conformational changes in the PR ligand-binding domain (LBD), thereby initiating subsequent gene regulatory cascades [20].

The best-evaluated inhibitors in terms of binding affinity were the molecules androstenedione, dihydrotestosterone, and 15α-hydroxyprogesterone, and thus the hydrophobic interactions were similar to those observed in the STR control for the amino acid residues Leu718, Leu721, and Phe778 and the hydrogen bond with Arg766 (Table 3). The required position and orientation of the Gln725 group were reinforced by the backbone carbonyl of Phe778, which, in turn, was fixed by van der Waals contacts with the progesterone A ring. These interactions corroborate the literature data for the classification of potential active constituents for PR inhibition [29,30].

The potential ligand androstenedione (see Figure 3) presented the best binding affinity value (−10.5 kcal/mol), followed by 17α-hydroxyprogesterone (−10.3 kcal/mol) and dihydrotestosterone (−10.3 kcal/mol), which are good PR inhibitors, with variations of ±0.50, ±0.70, and ±0.70 kcal/mol concerning STR, respectively. The conformation of inhibitor site conformation is influenced by the distance of their interactions with amino acid residues.

### 2.3. Biological Activity Prediction

Pa and Pi are the measures of the probability that a molecule is active or inactive, respectively. It is reasonable to assume that only these types of activities are revealed by molecules when Pa > Pi [31]. Consequently, if Pa ≥ 0.3, the molecule will probably present a positive result in experiments. Consequently, the probability of this molecule being analogous to known pharmaceutical agents is high. The activities originating from the descriptors of neighboring atoms in the chemical structures, based on the spectrum, are listed in Table 4.

Androstenedione, 15α-hydroxyprogesterone, and dihydrotestosterone were predicted to have biological activities: antineoplastic (BC), with a range of 0.535–0.354, and anticarcinogenic. Biological activity with Pa > Pi is considered probable when compared with the selected drugs.

The selected potential molecules showed structural similarity to the STR control, a steroid hormone that exerts a wide variety of biological effects in mammalian organisms. Steroids occupy an important place in fungi, and ergosterols (ergostane) are characteristic of both fungi and plants. Most ergosterol metabolites are cytotoxic and are active against many types of cancer cells [27].

### 2.4. Perspective of Obtaining Compounds with Antineoplastic Activity (BC) from Biotransformation by Fungi

In recent years, the chemical and pharmaceutical industries have realized that biotransformation is a crucial production technique for creating molecules with added value. The specialized transformation of a molecule into a new product with structural similarities using microorganisms, sometimes known as biological catalysts, is called biotransformation [32]. Microorganisms with numerous enzymes have been used as biological catalysts [33] in different steroid reactions. Oxidation, reduction, isomerization, hydrolysis, the addition of functional groups, and the creation of new carbon bonds are some of the categories under which these changes can be grouped [34,35]. To date, approximately 300 steroid drugs have been approved, and this number is increasing. Steroid pharmaceuticals are ranked among the most marketed medical products and represent the second largest category after antibiotics [36]. Bioconversion can be performed at a position of the steroid molecule that is hardly available for chemical agents; functionalization of the molecule can be performed regio- and stereospecifically; several reactions can be completed in one biotechnological step; and metabolic pathways can be constructed in a specific sequence in the newly generated strain. Thus, the biotransformation of progesterone has emerged as a cutting-edge method to facilitate the manufacture of a variety of steroid-derived compounds in the last few decades [37].

**Figure 4:**
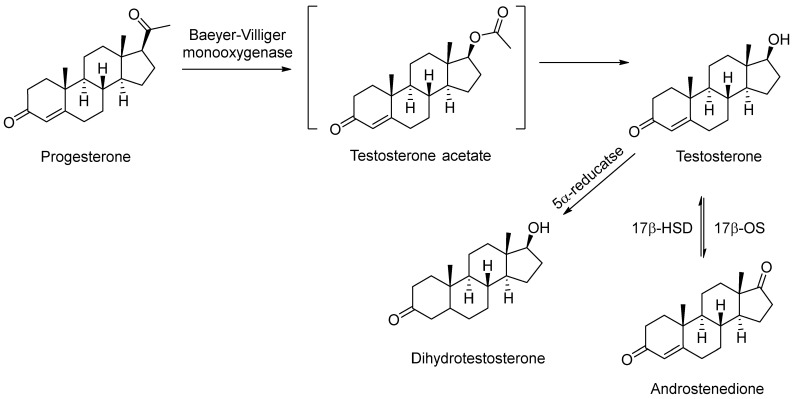
Bio-oxidation of progesterone from Baeyer–Villiger monooxygenase mechanisms.

According to Table 5, the biotransformation of progesterone by filamentous fungi commonly leads to the formation of androstenedione, either as a final product or as an intermediate in the process of D-lactonization of C17-ketones by the Baeyer–Villiger monooxygenase mechanisms (BVMOs) [38] (Figure 4), through the action of enzymes of the type 17β-hydroxysteroid dehydrogenase (17β-HSD). Androstenedione is a key intermediate in the body’s steroid metabolism, used as a precursor for several steroid substances, such as testosterone, estradiol, ethinyl estradiol, testolactone, progesterone, cortisone, cortisol, prednisone, and prednisolone [39]. Strains from *Aspergillus, Penicillium, Fusarium*, and other genera (Table 5) were capable of efficient progesterone formation using androstenedione.

On the other hand, the conversion to dihydrotestosterone from progesterone indicates the participation of the enzyme 5α-reductase produced by the fungus. The 5α-reductase activity has been well demonstrated in *F. oxysporum var. cubense, P. chrysogenum*, and *P. crustosum*, which were isolated from corn tortillas [40] or *P. decumbens* broth obtained from fermented pistachios and lemons (Table 5) [41]. The 5α-reductase enzyme converts Δ4-3-ketosteroids to 5α-3-ketosteroids in androgen-dependent tissue. In humans, 5α -reductase acts on a variety of androgen-responsive target tissues to mediate such diverse endocrine processes as male differentiation in the fetus and prostatic growth in adult men and plays a role in several endocrine abnormalities, including benign prostate hyperplasia, male pattern baldness, acne, and hirsutism [41]. In the study of steroids today, there is great interest in the mammalian steroid 5α-reductase, which converts the 3-keto-Δ4-steroids, testosterone, progesterone, and hydrocortisone to their respective 5α-dihydroderivatives. This interest focuses on finding and developing specific inhibitors for the 5α-reductase enzyme that is responsible for the in vivo conversion of testosterone to 5α-DHT, because enhanced levels in men have been implicated in several clinical disorders such as benign prostatic hyperplasia, male pattern baldness, and acne [40].

Hydroxylation involves the direct oxidation of a C–H bond to produce an alcohol. Bioconversions of 15α-hydroxylation from progesterone by the fungi *A. fumigatus, Didymosphera igniaria, F. cumorum, P. oxalicum* CBMAI 1996, and *Thanatephorus cucumeris* have been shown (Table 5). These reactions may occur at various points on the molecule, especially the hydroxylation of non-activated centers, which is difficult to achieve using classical chemical methods. Hydroxylation changes the polarity of steroids and influences their toxicity, excretion from the cell, and translocation via the cell envelope [42]. The hydroxylation of steroids is important because of their physiological role in mammalian organisms and for practical reasons [7].

**Table 5:** Species of fungi capable of biotransformation target products.

Fungus	Products Biotransformation from Progesterone			Ref.
	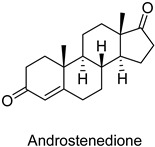	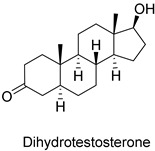	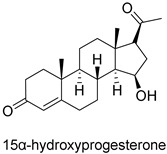	
*A. fumigatus*			**+**	[7]
*A. sojae* PTCC 5196	+			[35]
*A. tamarii*	+			[43]
*A. tamarii*	+			[44]
*A. terreus* MRC 200365	+			[35]
*B. felina* (CBMAI 738)	+			[45]
*Corynespora cassiicola* CBS 161.60	+			[46]
*Didymosphera igniaria*			**+**	[7]
*F. cumorum*			+	[7]
*F. oxysporum* SC1301	+			[47]
*F.* *oxysporum var. cubense*		+		[48]
*Fusarium sp.* (CBMAI 1830)	+			[45]
*H. werneckii*	+			[49]
*P. chrysogenum*		+		[40]
*P. citreo-viride*	+			[50]
*P. citreo-viride* ACCC 0402	+			[35]
*P. citrinum* (CBMAI 1186)	+			[45]
*P. crustosum*		+		[40]
*P. decumbens*		+		[41]
*P. oxalicum*CBMAI 1996			+	[51]
*P. oxalicum* (CBMAI 1185)	+			[45]
*Saprolignia hypogyna*	+			[7]
*T. arzianum* (CBMAI 1677)	+			[45]
*Talaromyces* sp. H4	+			[52]
*Thanatephorus cucumeris*			+	[53]
*Whetzelinia sclerotiorum*	+			[54]

+ Species capable of producing from the biotransformation of progesterone.

## 3. Materials and Methods

### 3.1. Obtainment, Design, and Geometric Optimization of Chemical Structures

The chemical structures described in the literature as substances identified by the metabolic expression and biotransformation processes of fungal species were selected [Supplementary Materials (SM)] for pharmacokinetic, toxicological, and ligand–receptor interaction studies in the identification of potential antineoplastic (breast cancer) agents. The structures were drawn in ChemDraw Ultra 12.0 software and saved in MDL Molfile (.mol) format [55]. Then, the geometric optimization of the three-dimensional (3D) structure was performed in ChemSketch software (ACD/Labs, v.12.01, 2016) by the Molecular Mechanics (MM+) method with the force field initially based on the CHARMM parameterization [56,57,58].

### 3.2. In Silico Evaluation of Pharmacokinetic and Toxicological Properties

Absorption, distribution, metabolism, excretion, and toxicity (ADMET) predictions were performed using the Discovery Studio v16 module, USA (2013). These properties are important in determining the success of the compound for human therapeutic use. Some important chemical descriptors correlate well with ADMET properties, such as polar surface area (PSA) as a primary determinant of fraction absorption and low molecular weight (MW) for oral absorption. Chemical compound distribution human body depends on factors such as penetration of the blood–brain barrier (Log BB), binding to plasma proteins and cytochrome P450 (CYP2D6), solubility, and intestinal absorption (AI) [14,59].

Toxicity prediction tests were performed using Discovery Studio 2015 software (Accelrys, Inc., San Diego, CA, USA), via the Toxicity Prediction by Komputer Assisted Technology (TOPKAT) function (BIOVIA, 2024). In this way, the module can predict the toxicity of chemicals based solely on their 2D molecular structure, using a variety of robust cross-validated quantitative structure–toxicity relationship (QSTR) models to assess specific toxicological parameters. The toxicological properties analyzed were carcinogenicity in rodents (female mice and female rats), Ames mutagenicity, skin irritation, and skin sensitization. Toxicity risk prediction calculations were performed via TOPKAT and measured the following parameters: oral LD50 (g/kg body weight), *Daphnia magna* (EC50 (mg/L)), the lowest dose at which toxic or adverse effects are observed (LOAEL) (g/kg body weight), and *Pimephales promelas* (LC50 (g/L). In addition, the carcinogenic potential was also predicted through the following parameters: TD50 (mg/kg body weight/mouse-day/rat) and the rat maximum tolerated dose—RMTD (mg/kg body weight).

### 3.3. Study of Molecular Docking Simulations

The structure of the hormone-bound human progesterone receptor (PR), elucidated by X-ray diffraction, was downloaded from the Protein Data Bank (https://www.rcsb.org/, accessed on 4 September 2024) with the PDB ID code 1A28 and a resolution of 1.80 Å [28]. Progesterone (STR) and estradiol (EST) were used as control ligands in the molecular docking studies. The ligand assignment of protonation and tautomeric states was performed with the Discovery Studio^®^ 2.0 program (2013), while the hydrogen atoms of the proteins were added with PROPKA using physiological pH (~7).

Molecular docking simulations were performed through the DockThor server (https://dockthor.lncc.br/v2/ (accessed on 4 September 2024)) [60]. The default parameters of the algorithm used were defined as follows: (1) 12 runs of docking conformations, (2) 500,000 evaluations per docking run, and (3) a population of 750 individuals. The protein–ligand docking score quality was evaluated based on the root mean square deviation (RMSD) between the overlay of binding mode best score (experimental pose vs. docking molecular pose).

### 3.4. Prediction of Biological Activity

Prediction of Biological Activity Spectra for Substances (PASS) was performed using the web server (http://www.pharmaexpert.ru/passonline (accessed on 16 October 2024)) [61]. In PASS, it is possible to discover the effects of a molecule based entirely on the molecular formula using multilevel neighboring descriptors of atoms, suggesting that biological activity is a function of its chemical structure [31,62]. Thus, only molecules with active potential for antineoplastic (breast cancer) and anticarcinogenic activities were selected.

## 4. Conclusions

In this study, 155 molecules described in the literature on the biotransformation processes of various fungal species were evaluated for their potential for anti-breast cancer activity. Molecules 55, 70, and 81 showed good pharmacokinetic profiles and low toxicity compared to the control groups. The in-silico data associated with molecular docking studies revealed the best binding affinity and similarity in the interactions of these molecules against the human progesterone receptor target. Thus, the results of biological activity spectrum prediction highlight the great potential to investigate the role of molecular descriptors in the attribution of anticancer activities. Thus, a deep understanding of the mechanism of anti-breast cancer activity is a better way that will undoubtedly lead to the development of new entities with improved and effective activity. The molecules identified in the study stand out for presenting great interaction and being potential inhibitors of the human progesterone receptor as an alternative for the treatment of breast cancer in experimental trials (in vitro and in vivo).

## Figures and Tables

**Table 1:** Oral bioavailability of TopHits3 molecules.

Molecules	PPB	Hepatotoxic	CYP2D6	Solubility	BBB	HIA
STR	true	false	false	2	1	0
Estradiol	true	false	false	2	1	0
Androstenedione	true	false	false	2	1	0
Dihydrotesteosterone	true	false	false	2	1	0
15α-Hydroxyprogesterone	true	false	false	2	2	0

BBB, blood–brain barrier; 0 (very high penetrance); 1 (high); 2 (medium); 3 (low); 4 (very low); HIA, human intestinal absorption (acceptable range: 0–2, where 0 is a good absorption); aqueous solubility (acceptable range: 0–3, where 3 is a good solubility); cytochrome P450 (CYP450) 2D6 inhibition (false—non-inhibitor; true—inhibitor); PPB, plasma protein binding (false—does not bind to plasma proteins; true—binds to plasma proteins) [15].

**Table 2:** Results of computational toxicity parameters in animal models, prediction of toxicity risk, and tolerated dose of the carcinogenic potency of TopHits3.

	Computational Toxicological Parameters						Prediction of Toxicity Risk					Tolerated Dose of Carcinogenic Potency		
Molecules							*Pimephales promelas*	*Daphnia magna*	Rat			Rat	Mouse	Rat
	Female/Male		Ames	Skin		Ocular			Oral	Chronic	Inhalation	(Body Weight/Day)		
	*mouse*	*rat*	*Mutagenicity*	*Irritancy*	*Sensitization*	*Irritation*	*LC_50_* (g/L)	*EC_50_* (mg/L)	*LD_50_* (g/kg)	*LOAEL* (g/kg)	*LC_50_* (mg/m^3^/h)	*TD_50_* (mg/kg)		*RMTD* (g/kg)
STR	C+	C+	M-	+++	+	++	0.001	1.043	2.15	0.015	6346.37	7.758	26.253	0.084
EST	C+	C+	M-	-	++++	++++	0.015	4.917	0.537	0.063	6790.71	1.159	22.564	0.229
Androstenedione	C+	C-	M-	+++	+	++	0.002	1.173	1.883	0.017	6169.48	7.161	11.910	0.064
Dihydrotesteosterone	C+	C-	M-	+++	+	++	0.050	9.500	2.365	0.025	5738.41	0.051	8.505	0.094
15α-Hydroxyprogesterone	C+	C-	M-	+++	+	++++	0.014	5.531	3.836	0.011	3741.09	0.475	39.463	0.046

MF: mouse female; RF: rat female; AM: Ames mutagenicity; SI: skin irritancy; SS: skin sensitization; OI: ocular irritancy; OR: oral rate; LD50: median lethal dose; FM: Fathead Minnow (short-term toxicity to fish *Pimephales promelas*); LC50: exposure concentration of a toxic substance lethal to half of the animals tested; DM: Daphnia magna; EC50: half maximal effective concentration (effective concentration of a substance causing adverse effects in 50% of the population tested—Daphnia magna); RCL: rat chronic LOAEL (lowest level of adverse effect observed); RI: rat inhalation; RMTD: rat maximum tolerated dose; TD50: carcinogenic potency value; - (none); C (single carcinogen); C+ (multi-carcinogen); C- (non-carcinogen); M- (non-mutagen); + (weak); ++ (mild); +++ (moderate); ++++ (severe).

**Table 3:** Binding affinity and main interactions in the PR.

Ligand	Binding Affinity	H-Bonds	Hydrophobic Interactions
STR	−11.0	Arg766	Leu715, Leu718, Leu721, Phe778, Leu797 and Tyr890
Androstenedione	−10.5	Gln725, Arg766	Leu721, Val760, Leu763, Phe778, Leu797 and Leu887
Dihydrotestosterone	−10.3	Gln725, Leu763, Arg766	Leu715, Leu718, Asn719, Phe778 and Phe794
15α-Hydroxyprogesterone	−10.3	-	Leu718, Leu721, Met759 and Tyr890

**Table 4:** Biological activity prediction for TopHits3.

Molecules	PASS		
	Pa ^1^	Pi ^2^	Biological Activity
STR	0.270	0.064	Antineoplastic (breast cancer)
	0.299	0.058	Anticarcinogenic
Estradiol	0.542	0.015	Antineoplastic (breast cancer)
	0.456	0.024	Anticarcinogenic
Androstenedione	0.505	0.018	Antineoplastic (breast cancer)
	0.414	0.029	Anticarcinogenic
Dihydrotestosterone	0.535	0.016	Antineoplastic (breast cancer)
15α-Hydroxyprogesterone	0.354	0.041	Antineoplastic (breast cancer)
	0.450	0.024	Anticarcinogenic

## Data Availability

The original contributions presented in this study are included in the article/Supplementary Materials. Further inquiries can be directed at the corresponding author.

## Supplementary Materials

The following supporting information can be downloaded at: https://www.mdpi.com/article/10.3390/ph18020136/s1. Figure S1. RMSD representation of the crystallographic ligand (green) and best docking pose (cyan) in the RP. Table S1. Obtaining, Designing, and Geometric Optimization of Chemical Structures. Table S2. In Silico Evaluation of Pharmacokinetic and Toxicological Properties.
