# Supplementary material for: Identification of Novel Progesterone Receptor (PR) Inhibitors (Homo sapiens) from Metabolites of Biotransformation Fungal: A Bioinformatics Approach
Source: Pharmaceuticals (Basel). 2025 Jan 21;18(2):136. doi: 10.3390/ph18020136 (PMC11858247; doi:10.3390/ph18020136)
Supplement: Supplementary file 1 [file pharmaceuticals-18-00136-s001.zip › pharmaceuticals-3366564-supplementary.pdf]

Supplementary Materials:

Identification of Novel Progesterone Receptor (PR) Inhibitors (*Homo sapiens*) from Metabolites of Biotransformation Fungal: A Bioinformatics Approach

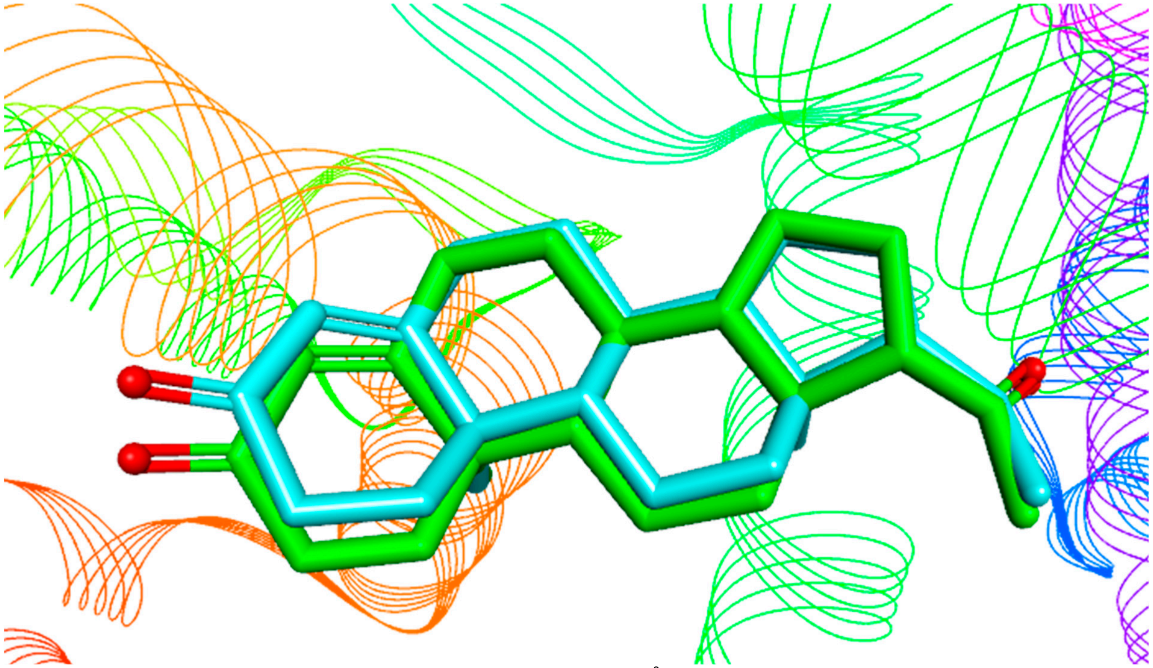

RMSD= 0.58 Å

Δ*G* = −11.92 Kcal/mol

Figure S1. RMSD representation of the crystallographic ligand (green) and best docking pose (cyan) in the RP.

Table S1. Obtaining, Designing, and Geometric Optimization of Chemical Structures.

Index	Name	ADMET_Solubility	ADMET_Level	AlogP98	ADMET_BBB	ADMET_BBB_Level	ADMET_EXT_CYP2D6	ADMET_EXT_CYP2D6#Prediction
1	5994	−5.719	2	0	0.492	1	−2.1115	false
2	5757	−4.341	2	0	0.373	1	−1.4357	false
3	152125	−4.366	.2	0	−0.151	2	−4.71632	false
4	222865	−5.313	2	0	0.35	1	−0.905942	false
5	67080759	−4.156	2	0	−0.225	2	−2.84779	false
6	225	−4.869	2	0	0.352	1	−1.59881	false
7	92810	−5.57	2	0	0.439	1	−1.07074	false
8	6013	−4.75	2	0	0.316	1	−2.03452	false
9	6128	−5.463	2	0	0.403	1	−1.7373	false
10	13472	−5.442	2	0	0.396	1	−1.80511	false
11	13769	−5.219	2	0	0.166	1	−4.69736	false
12	92145	−5.625	2	0	0.348	1	−2.44975	false
13	92750	−4.213	2	0	−0.199	2	−2.36225	false
14	108106	−3.972	3	0	−0.288	2	−2.27573	false
15	15	−4.194	2	0	−0.206	2	−3.5504	false
16	16	−2.925	3	0	−0.876	3	−5.15703	false
17	17	−3.376	3	0	−0.834	3	−5.46572	false
18	20	−3.342	3	0	−0.742	3	−5.26967	false
19	21	−3.393	3	0	−0.361	2	−3.77907	false
20	22	−5.313	2	0	0.35	1	−0.905942	false
21	24	−4.75	2	0	0.316	1	−2.03452	false
22	25	−5.57	2	0	0.439	1	−1.07074	false
23	26	−5.036	2	0	0.38	1	−3.27257	false
24	27	−2.887	3	0	−0.89	3	−5.35167	false
25	28	−4.222	2	0	−0.199	2	−2.36225	false
26	29	−4.347	2	0	−0.158	2	−4.39241	false
27	30	−3.096	3	0	−0.821	3	−7.49854	false
28	31	−4.364	2	0	−0.151	2	−4.71632	false
29	32	−4.366	2	0	−0.151	2	−4.71632	false
30	33	−4.213	2	0	−0.199	2	−2.36225	false
31	34	−2.942	3	0	−0.869	3	−5.14447	false

32	35	−4.222	2	0	−0.199	2	−2.36225	false
33	36	−4.169	2	0	−0.175	2	−1.22581	false
34	37	−4.213	2	0	−0.199	2	−2.36225	false
35	38	−2.942	3	0	−0.869	3	−5.14447	false
36	39	−3.395	3	0	−0.827	3	−4.69102	false
37	40	−5.037	2	0	−0.168	2	−2.67884	false
38	41	−4.213	2	0	−0.199	2	−2.36225	false
39	42	−5.463	2	0	0.403	1	−1.7373	false
40	43	−5.265	2	0	0.493	1	−1.62759	false
41	44	−3.342	3	0	−0.375	2	−3.21156	false
42	45	−4.213	2	0	−0.199	2	−2.36225	false
43	46	−4.366	2	0	−0.151	2	−4.71632	false
44	47	−4.564	2	0	−0.046	2	−1.56037	false
45	48	−4.213	2	0	−0.199	2	−2.36225	false
46	49	−2.595	3	0	−1.268	3	−5.87183	false
47	50	−5.117	2	0	0.286	1	−3.38771	false
48	51	−4.213	2	0	−0.199	2	−2.36225	false
49	52	−4.366	2	0	−0.151	2	−4.71632	false
50	53	−4.283	2	0	−0.178	2	−3.25366	false
51	54	−5.625	2	0	0.348	1	−2.44975	false
52	55	−5.463	2	0	0.403	1	−1.7373	false
53	56	−4.75	2	0	0.316	1	−2.03452	false
54	57	−5.719	2	0	0.492	1	−2.1115	false
55	58	−6.642	1	0	1.092	0	−0.978174	false
56	59	−5.123	2	0	0.44	1	−1.43717	false
57	60	−4.667	2	0	0.442	1	−0.513618	false
58	61	−5.55	2	0	0.431	1	−2.36163	false
59	62	−5.265	2	0	0.493	1	−1.62759	false
60	63	−5.233	2	0	0.477	1	−1.77128	false
61	64	−5.265	2	0	0.493	1	−1.62759	false
62	65	−4.366	2	0	−0.151	2	−4.71632	false
63	66	−4.364	2	0	−0.151	2	−4.71632	false
64	67	−3.096	3	0	−0.821	3	−7.49854	false

65	68	−2.978	3	0	−0.861	3	−5.71571	false
66	69	−4.213	2	0	−0.199	2	−2.36225	false
67	70	−4.222	2	0	−0.199	2	−2.36225	false
68	71	−4.219	2	0	−0.199	2	−2.36225	false
69	72	−2.887	3	0	−0.89	3	−5.35167	false
70	73	−2.881	3	0	−0.89	3	−4.77961	false
71	74	−5.719	2	0	0.492	1	−2.1115	false
72	75	−4.222	2	0	−0.199	2	−2.36225	false
73	76	−2.941	3	0	−0.869	3	−3.68099	false
74	77	−5.036	2	0	0.38	1	−3.27257	false
75	78	−3.032	3	0	−0.842	3	−6.03507	false
76	79	−2.887	3	0	−0.89	3	−5.35167	false
77	80	−5.313	2	0	0.35	1	−0.905942	false
78	81	−4.6	2	0	0.263	1	−0.60424	false
79	82	−3.376	3	0	−0.375	2	−3.2147	false
80	83	−3.78	3	0	−0.63	3	−3.83415	false
81	84	−4.213	2	0	−0.199	2	−2.36225	false
82	85	−4.283	2	0	−0.178	2	−3.25366	false
83	86	−4.213	2	0	−0.199	2	−2.36225	false
84	87	−3.887	3	0	−0.915	3	−6.81405	false
85	88	−3.369	3	0	−0.375	2	−4.40755	false
86	89	−5.595	2	0	0.251	1	−1.83332	false
87	90	−3.78	3	0	−0.63	3	−3.83415	false
88	91	−3.498	3	0	−0.326	2	−5.56563	false
89	92	−3.411	3	0	−0.354	2	−4.10298	false
90	93	−3.461	3	0	−0.333	2	−3.30939	false
91	94	−5.643	2	0	0.3	1	−3.30518	false
92	95	−4.213	2	0	−0.199	2	−2.36225	false
93	96	−4.65	2	0	0.275	1	−2.59434	false
94	97	−5.093	2	0	0.273	1	−1.69427	false
95	98	−5.491	2	0	0.306	1	−3.90099	false
96	99	−4.75	2	0	0.316	1	−2.03452	false
97	100	−4.366	2	0	−0.151	2	−4.71632	false

98	101	−5.233	2	0	0.477	1	−1.77128	false
99	102	−4.283	2	0	−0.178	2	−3.25366	false
100	103	−4.778	2	0	0.479	1	−1.11133	false
101	104	−5.265	2	0	0.493	1	−1.62759	false
102	105	−4.366	2	0	−0.151	2	−4.71632	false
103	106	−4.364	2	0	−0.151	2	−4.71632	false
104	107	−3.096	3	0	−0.821	3	−7.49854	false
105	108	−2.96	3	0	−0.868	3	−5.72827	false
106	109	−4.202	2	0	−0.206	2	−3.5504	false
107	110	−4.2	2	0	−0.206	2	−3.5504	false
108	111	−4.202	2	0	−0.206	2	−3.5504	false
109	113	−4.213	2	0	−0.199	2	−2.36225	false
110	114	−2.881	3	0	−0.89	3	−4.77961	false
111	115	−4.219	2	0	−0.199	2	−2.36225	false
112	116	−2.887	3	0	−0.89	3	−5.35167	false
113	117	−3.212	3	0	−0.779	3	−6.2154	false
114	118	−2.881	3	0	−0.89	3	−4.77961	false
115	119	−3.026	3	0	−0.842	3	−6.03507	false
116	120	−3.032	3	0	−0.842	3	−6.03507	false

Table S2. In Silico Evaluation of Pharmacokinetic and Toxicological Properties.

Index	Hepatotoxic#Prediction	Absor-tion_Level	PPB_#Prediction	AlogP98	Fathead_Minnow_LC50	Fathead_Minnow_LC50_Unit	Daphnia_EC50	Daphnia_EC50_Unit
1	false	0	true	3.86	0.00103555	g/L	1.04304	mg/L
2	false	0	true	3.838	0.0153743	g/L	4.91734	mg/L
3	false	0	true	2.846	0.0138238	g/L	4.58638	mg/L
4	true	0	true	3.402	0.00892806	g/L	2.12638	mg/L
5	false	0	true	2.606	0.0222994	g/L	6.49049	mg/L
6	false	0	true	3.588	0.0364821	g/L	5.33276	mg/L
7	true	0	true	3.688	0.00441519	g/L	1.88845	mg/L
8	false	0	true	3.473	0.0116797	g/L	3.29339	mg/L
9	false	0	true	3.573	0.00209271	g/L	1.17372	mg/L
10	false	0	false	3.55	0.00218592	g/L	1.20069	mg/L
11	false	0	false	3.263	0.00172396	g/L	1.5856	mg/L
12	false	0	true	3.852	0.00052731	g/L	0.378245	mg/L
13	false	0	true	2.69	0.0137023	g/L	5.53132	mg/L
14	false	0	true	2.404	0.0277915	g/L	6.2514	mg/L
15	false	0	false	2.667	0.0143262	g/L	5.66379	mg/L
16	false	0	false	1.565	0.0664224	g/L	8.875	mg/L
17	false	0	false	1.523	0.0367845	g/L	9.0361	mg/L
18	false	0	false	1.999	0.0413882	g/L	7.06219	mg/L
19	false	0	false	2.348	0.0569508	g/L	4.3632	mg/L
20	true	0	true	3.402	0.00892806	g/L	2.12638	mg/L
21	false	0	true	3.473	0.0116797	g/L	3.29339	mg/L
22	true	0	true	3.689	0.00441519	g/L	1.88845	mg/L
23	true	0	true	3.678	0.00980441	g/L	2.55746	mg/L
24	false	0	true	1.521	0.0683224	g/L	7.33339	mg/L
25	false	0	true	2.69	0.0137023	g/L	5.53132	mg/L
26	false	0	false	2.823	0.0144532	g/L	4.69621	mg/L
27	false	0	true	1.744	0.0535811	g/L	6.38773	mg/L
28	false	0	true	2.846	0.0138238	g/L	4.58638	mg/L
29	false	0	true	2.846	0.0138238	g/L	4.58638	mg/L
30	false	0	true	2.69	0.0137023	g/L	5.53132	mg/L

31	false	0	true	1.589	0.0634417	g/L	7.14027	mg/L
32	false	0	true	2.69	0.0137023	g/L	5.53132	mg/L
33	false	0	true	2.769	0.00767104	g/L	8.77047	mg/L
34	false	0	true	2.69	0.0137023	g/L	5.53132	mg/L
35	false	0	true	1.589	0.0634417	g/L	7.14027	mg/L
36	false	0	true	1.546	0.035174	g/L	6.91311	mg/L
37	false	0	true	3.07	0.000607962	g/L	0.284887	mg/L
38	false	0	true	2.69	0.0137023	g/L	5.53132	mg/L
39	false	0	true	3.573	0.00209271	g/L	1.17372	mg/L
40	false	0	true	4.046	0.00422907	g/L	4.55763	mg/L
41	false	0	true	2.303	0.0586922	g/L	4.37871	mg/L
42	false	0	true	2.69	0.0137023	g/L	5.53132	mg/L
43	false	0	true	2.846	0.0138238	g/L	4.58638	mg/L
44	false	0	true	3.188	0.00485866	g/L	10.5593	mg/L
45	false	0	true	2.69	0.0137023	g/L	5.53132	mg/L
46	false	0	true	1.184	0.0326013	g/L	12.4684	mg/L
47	false	0	true	4.441	0.0049386	g/L	14.2641	mg/L
48	false	0	true	2.69	0.0137023	g/L	5.53132	mg/L
49	false	0	true	2.846	0.0138238	g/L	4.58638	mg/L
50	false	0	true	2.758	0.0127235	g/L	5.38566	mg/L
51	false	0	true	3.852	0.00052731	g/L	0.378245	mg/L
52	false	0	true	3.573	0.00209271	g/L	1.17372	mg/L
53	false	0	true	3.473	0.0116797	g/L	3.29339	mg/L
54	false	0	true	3.86	0.00103555	g/L	1.04304	mg/L
55	false	0	true	4.917	9.87E−05	g/L	0.444488	mg/L
56	false	0	true	3.874	0.0180501	g/L	7.35536	mg/L
57	false	0	true	4.06	0.00766233	g/L	7.15424	mg/L
58	true	0	true	3.665	0.001412	g/L	1.85606	mg/L
59	false	0	true	4.046	0.00422907	g/L	4.55763	mg/L
60	true	0	true	3.994	0.00155372	g/L	2.82628	mg/L
61	false	0	true	4.046	0.00422907	g/L	4.55763	mg/L
62	false	0	true	2.846	0.0138238	g/L	4.58638	mg/L
63	false	0	true	2.846	0.0138238	g/L	4.58638	mg/L

64	false	0	true	1.744	0.0535811	g/L	6.38773	mg/L
65	true	0	true	1.615	0.0616701	g/L	7.44157	mg/L
66	false	0	true	2.69	0.0137023	g/L	5.53132	mg/L
67	false	0	true	2.69	0.0137023	g/L	5.53132	mg/L
68	false	0	true	2.69	0.0137023	g/L	5.53132	mg/L
69	false	0	true	1.521	0.0683224	g/L	7.33339	mg/L
70	false	0	true	1.521	0.0683224	g/L	7.33339	mg/L
71	false	0	true	3.86	0.00103555	g/L	1.04304	mg/L
72	false	0	true	2.69	0.0137023	g/L	5.53132	mg/L
73	false	0	true	1.589	0.0634417	g/L	7.14027	mg/L
74	true	0	true	3.678	0.00980441	g/L	2.55746	mg/L
75	false	0	true	1.677	0.0576393	g/L	6.55747	mg/L
76	false	0	true	1.521	0.0683224	g/L	7.33339	mg/L
77	true	0	true	3.402	0.00892806	g/L	2.12638	mg/L
78	false	0	true	3.301	0.0498809	g/L	9.49884	mg/L
79	false	0	true	2.303	0.0586922	g/L	4.92124	mg/L
80	false	0	false	1.755	0.0369426	g/L	12.2301	mg/L
81	false	0	true	2.69	0.0137023	g/L	5.53132	mg/L
82	false	0	true	2.758	0.0127235	g/L	5.38566	mg/L
83	false	0	true	2.69	0.0137023	g/L	5.53132	mg/L
84	false	0	true	2.174	0.0239031	g/L	1.48677	mg/L
85	false	0	true	2.303	0.0586922	g/L	4.37871	mg/L
86	false	0	true	3.995	0.000143397	g/L	0.194804	mg/L
87	false	0	false	1.755	0.0369426	g/L	12.2301	mg/L
88	false	0	true	2.459	0.049515	g/L	4.5613	mg/L
89	false	0	true	2.371	0.0544995	g/L	4.2634	mg/L
90	false	0	true	2.438	0.0506614	g/L	3.89317	mg/L
91	false	0	true	3.697	0.000924226	g/L	1.19467	mg/L
92	false	0	true	2.69	0.0137023	g/L	5.53132	mg/L
93	false	0	true	3.338	0.0135312	g/L	3.20791	mg/L
94	false	0	true	3.152	0.00331126	g/L	1.27906	mg/L
95	false	0	true	3.717	0.000610899	g/L	0.368427	mg/L
96	false	0	true	3.473	0.0116797	g/L	3.29339	mg/L

97	false	0	true	2.846	0.0138238	g/L	4.58638	mg/L
98	true	0	true	3.994	0.00155372	g/L	2.82628	mg/L
99	false	0	true	2.758	0.0127235	g/L	5.38566	mg/L
100	true	0	true	4.18	0.00174435	g/L	4.24579	mg/L
101	false	0	true	4.046	0.00422907	g/L	4.55763	mg/L
102	false	0	true	2.846	0.0138238	g/L	4.58638	mg/L
103	false	0	true	2.846	0.0138238	g/L	4.58638	mg/L
104	false	0	true	1.744	0.0535811	g/L	6.38773	mg/L
105	false	0	false	1.592	0.0644961	g/L	7.62194	mg/L
106	false	0	false	2.667	0.0143262	g/L	5.66379	mg/L
107	false	0	false	2.667	0.0143262	g/L	5.66379	mg/L
108	false	0	false	2.667	0.0143262	g/L	5.66379	mg/L
109	false	0	true	2.69	0.0137023	g/L	5.53132	mg/L
110	false	0	true	1.521	0.0683224	g/L	7.33339	mg/L
111	false	0	true	2.69	0.0137023	g/L	5.53132	mg/L
112	false	0	true	1.521	0.0683224	g/L	7.33339	mg/L
113	false	0	true	1.879	0.0462496	g/L	7.01443	mg/L
114	false	0	true	1.521	0.0683224	g/L	7.33339	mg/L
115	false	0	true	1.677	0.0576404	g/L	6.55792	mg/L
116	false	0	true	1.677	0.0576404	g/L	6.55792	mg/L
